# ABO Blood Groups and Its Association with Oral Cancer, Oral Potentially Malignant Disorders and Oral Submucous Fibrosis– A Systematic Review and Meta-Analysis

**DOI:** 10.31557/APJCP.2021.22.6.1703

**Published:** 2021-06

**Authors:** Abhinav Singh, Bharathi M Purohit

**Affiliations:** 1 *Department of Dentistry, Regional Training Centre for Oral Health Promotion, All India Institute of Medical Sciences (AIIMS) Bhopal, India. *; 2 *Division of Public Health Dentistry, Centre for Dental Education and Research, WHO Collaborating Centre for Oral Health Promotion, All India Institute of Medical Sciences, New Delhi, India. *

**Keywords:** ABO blood group system, oral cancer, potentially malignant disorders, oral submucous fibrosis

## Abstract

**Objective:**

The study aimed to evaluate the association between ABO blood groups and oral cancer, other potentially malignant disorders (OPMD), and oral submucous fibrosis (OSMF).

**Materials and methods:**

A search was conducted in Medline, Cochrane databases, Google Scholar, Scopus, Web of Science and Directory of Open Access Journals (DOAJ) for studies evaluating ABO blood groups as risk factors for oral cancer and OPMD among cases and controls. The PRISMA guidelines were followed for the meta-analysis. Participants included patients with oral cancer, and OPMD diagnosed using histopathologic investigations. Sub-group analysis was conducted to evaluate the association between blood groups and OSMF. Quality was evaluated using the Risk of Bias Assessment tool. Fixed effects model was used to assess the odds ratio for the association.

**Results:**

There were 1,352, 414, and 299 cases of oral cancer, OPMD, and OSMF and 11,699, 7,382 and 7,307 controls for analysis respectively. Blood group A was significantly associated with both oral cancer (Odds ratio: 1.27 [95% CI, 1.10, 1.47], P= 0.001) and OPMD (Odds ratio: 1.33 [95% CI, 1.01, 1.47], P= 0.04). No association was noted between blood group B and AB with oral cancer, OPMD, and OSMF. Blood group O was significantly associated with lower chances of oral cancer (Odds ratio: 0.81 [95% CI, 0.71, 0.93], P= 0.002).

**Conclusion:**

Meta-analysis suggests blood group A has a greater risk for developing oral cancer and OPMD. Blood group O was associated with lower chances of oral cancer. No association was noted between ABO blood group system with OSMF.

## Introduction

Oral cancer is one of the widely prevalent malignant diseaseand a major public health problem. There are an estimated 657,000 new cases of oral cancers each year, and more than 330,000 deaths (WHO, 2020).Oral and pharyngeal cancer, grouped together is the 6^th^ most common cancer in the world and the most common head and neck cancer in India (Warnakulasuriya, 2009; ICMR and NCDIR, 2020). Majority of the global incidence of oral cancer occurs in developing countries; half of those cases are in South Asia. India reports for more than one-fourth of all oral cancer deathswith an age-standardized incidence rate of 12.6 per lakh population and is estimated to record a 12% rise in cancer by 2025 (Sankaranarayanan et al., 2015; ICMR and NCDIR, 2020).

World Health Organization in 2005 suggested a term oral potentially malignant disorders (OPMD) and defined it as the risk of malignancy being present in a lesion or condition either at the time of initial diagnosis or at a future date (Wanakulasuriya et al., 2007). Oral hypoplasia, dysplasia, leukoplakia, erythroplakia and oral submucous fibrosis were included under broad term OPMD. OPMD showed a high risk of malignant transformation and the observed rates ranged from 0.6% to 36% (Shah et al., 2008; Dadhich et al., 2015).

The increasing number of oral malignancy and potentially malignant disorders related morbidity and mortality has been related to its multi-factorial aetiology; besides lifestyle and environmental factors, genetics and heredity have important role in causing cancer. Tobacco use and alcohol consumption account for more than 90 percent of oral cancers. Approximately 5 to 10 percent of cases are entirely hereditary (Warnakulasuriya, 2009). The ABO blood group is one of the genetic factors that has been hypothesized as the risk factors for different type of cancers.

The possibility of association between ABO blood groups and malignancy was first explored by Anderson and Haas (Anderson and Haas, 1984). Association of blood Group A in various cancers, including gastric cancers, breast cancers, pancreatic cancers, skin cancers, ovary and oesophageal cancers has been reported in various cohort studies and systematic reviews (Vasan et al., 2016; Mao et al., 2019; Miao et al., 2014; Celic et al., 2019; Huang et al., 2015). The association between ABO blood type with oral cancer and other potentially malignant disorders has also been studied but has yet not been established as risk factors due to contradictory findings (Gupta et al., 2020; Mortazavi et al., 2014; Jaleel and Nagarajappa, 2012; Jalili et al., 2018; Jamil et al., 2006; Kumari et al., 2017; Oroei, 2019; Poornima et al., 2018; Shishodia et al., 2018; Singh et al., 2014; Bhateja et al., 2014).

Blood group antigens in humans are present on the surface of red blood cells and epithelial cells of tissues such as mucosa and body fluids. The mechanism hypothesized behind the association between blood group A and carcinomas is that the carcinoma cells produce an antigen immunologically related to blood group A which predominantly in O group individuals may have a shielding effect by checking the growth and spread of the tumor. People with blood groups A and AB lack antibodies to A and are more prone to develop these carcinomas (Beckman and Angqvist, 1987; Henderson et al., 1993). Loss of expression of A or B antigens in more than 80% of cases of OSCC and OPMD have also been reported by immune-histochemical evaluations (Dabelsteen and Gao, 2015).

Numerous studies have been conducted to assess ABO blood group and its association with oral cancer, other potentially malignant disorders, and oral submucous fibrosis (Gupta et al., 2020; Mortazavi et al., 2014; Jaleel and Nagarajappa, 2012; Jalili et al., 2018; Jamil et al., 2006; Kumari et al., 2017; Oroei, 2019; Poornima et al., 2018; Shishodia et al., 2018; Singh et al., 2014; Bhateja et al., 2014). However, evidence from these studies remains inconclusive. This paper is a meta-analysis which aims to evaluate whether any ABO blood group types have a higher predisposition for oral cancer, OPMD, and OSMF. The study aims to determine the association between oral cancer, OPMD, and OSMF. The Population Exposure Comparison Outcome (PECO) question was population: participants were patients with oral cancer, OPMD, and OSMF diagnosed using histopathologic investigations; exposure to the risk factor: ABO blood grouping; comparison: healthy population with no oral cancer, OPMD, and OSMF; and outcome: prevalence of oral cancer, OPMD, and OSMF.

## Materials and Methods

*Inclusion criteria*

Inclusion and exclusion criteria were determined before the literature search. All observational studies including case-control, prospective and retrospective studies evaluating the association between ABO blood group among patients with oral cancer, oral potentially malignant disorders (OPMD), and oral submucous fibrosis (OSMF) as cases with healthy participants as controls were included. Participants were patients confirmed using histopathologic investigations. Exposure to the risk factor was ABO blood grouping among cases and controls. Outcome was the prevalence of oral cancer, OPMD, and OSMF. In vitro experiments or experiments involving laboratory animals were excluded. 

*Search strategy*

A search was conducted in Medline, Cochrane databases, Google Scholar, Scopus, Web of Science and Directory of Open Access Journals (DOAJ) for studies through May 2021. PRISMA guidelines were followed for the meta-analysis. Literature search keywords were: ABO blood group system AND oral cancer OR precancer OR oral sub mucous fibrosis. Boolean operators used with keywords are attached as an appendix. Studies were selected independently by two investigators (AS and BP). The title and abstracts were pre-screened to decide if the studies would be retrieved in full and to exclude ineligible studies. Retrieved articles were read before they were included in the review. Differences between the two investigators were resolved by discussion. A third person with subject expertise had been pre-approved by the two investigators in case of lack of consensus. References in the selected papers were manually reviewed and retrieved if they were relevant. The articles were searched using English keywords. No restrictions were placed on the publication language. Grey literature was searched through unpublished articles and manual searching of non-indexed journals at the institutional library at All India Institute of Medical Sciences (AIIMS), New Delhi (WHO Collaborating Centre for Oral Health Promotion) and AIIMS, Bhopal and through abstracts, conference presentations, online clinical registries including results of completed but unpublished trials for inclusion in the review. 

*Data extraction and quality assessment*

Dichotomous outcome data for the absence or presence of oral cancer, oral potentially malignant disorders (OPMD), and oral submucous fibrosis (OSMF) was extracted from the included studies in a pilot tested worksheet. The following variables were extracted from each study: first author’s name, publication year, country of origin, study design, numbers of cases and controls in different ABO blood types. Outcome data were extracted independently by the two investigators (AS and BP) using guidelines published by the Cochrane Collaboration (The Cochrane Collaboration, 2011). Differences between the two investigators were resolved by discussion. Characteristics of the studies that were included in the meta-analysis are presented in Table 1. Quality was evaluated by both the investigators using the Risk of Bias Assessment tool for Non-randomized Studies (Kim et al., 2013). Six domains (participant selection, confounding variables, exposure measurement, attrition for prospective studies, incomplete outcome data, and selective outcome reporting) were evaluated according to a low, moderate, and high risk of bias (Table 3). Attrition was to be reported only for prospective studies. 

Certainty of evidence was evaluated using GRADE assessment (a systematic approach to rating the certainty of evidence in systematic reviews and other evidence syntheses) (The GRADE Handbook, 2013). Explicit consideration was given to the type of included studies, risk of bias, consistency, directness of evidence, precision of results, risk of publication bias, magnitude of the effect, and influence of residual plausible confounding factors. The GRADE approach assesses the quality of a body of evidence as high, moderate, low, or very low.

*Analysis*

A systematic review was conducted using Cochrane Program Review Manager Version 5. A fixed effects model was used to assess the association between ABO blood groups and oral cancer and OPMD. Blood groups are fixed in an individual and are the independent variables, the dependent variable changes in response to the level of independent variables. Fixed effect model was used as the effect size was assumed to be fixed for different blood groups, irrespective of study location. Sub-group analysis was conducted to evaluate the association between blood groups and OSMF. The odds ratio with 95% confidence interval was calculated to evaluate the association levels. Certainty of evidence was assessed using GRADE analysis. Statistical significance was set at p < 0.05. 

## Results

**Figure 1:**
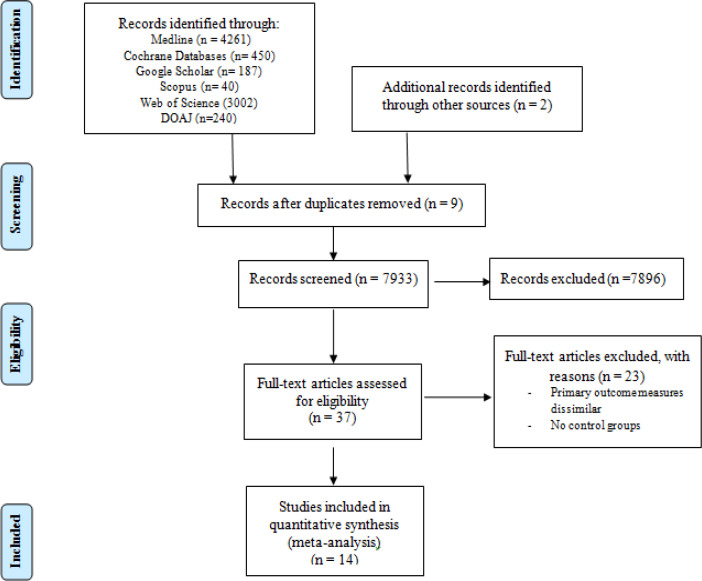
Flow Diagram of Study Selection Process

There were 37 potentially relevant publications, of which 14 were included in the review (Figure 1). Characteristics of the included studies are described in Table 1. There were 1352, 414 and 299 cases of oral cancer, OPMD, and OSMF respectively. The number of controls available for analysis with cases of oral cancer, OPMD, and OSMF were 11,699, 7,382 and 7,307 respectively. Table 2 presents the quality of studies that were included in the analysis. A low-to-moderate risk of bias was present for confounding variables. Additionally, a low risk of bias was present for incomplete outcome data and selective outcome reporting. Attrition levels were not recorded as there were no prospective studies.

**Figure 2.**
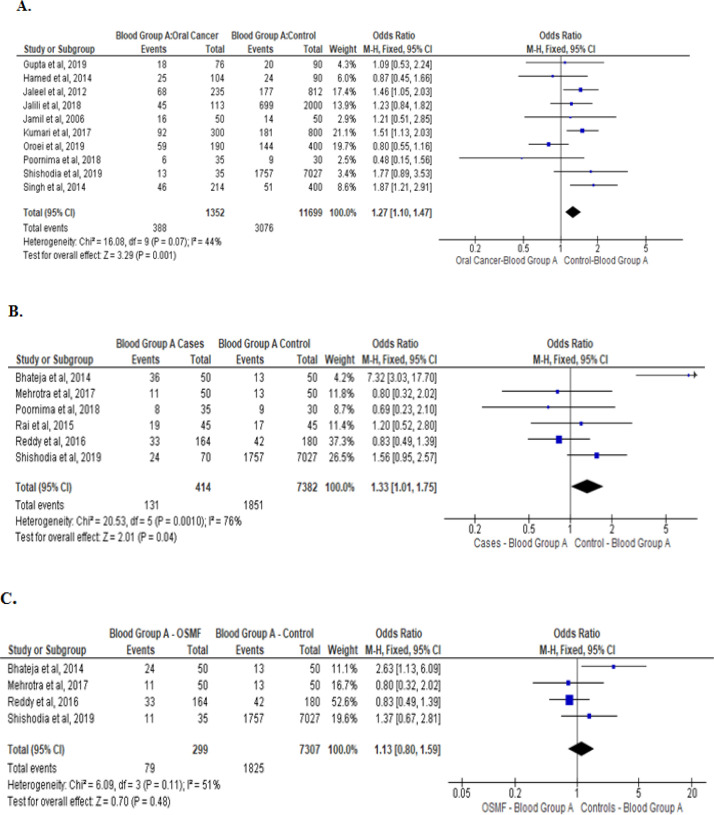
Type A Blood Group and Its Association between: Oral cancer patients with healthy controls (A) OPMD patients versus healthy controls (B), and OSMF patients versus healthy controls (C)

Meta-analysis of studies that evaluated the asociation of type A blood group withoral cancer, OPMD, and OSMF are presented in Figure 2. Blood group A was significantly associated with both oral cancer(Odds ratio: 1.27[95% CI, 1.10, 1.47], P= 0.001)and OPMD (Odds ratio: 1.33[95% CI, 1.01, 1.47], P= 0.04). However, no such association was noted between blood group A and OSMF. Sub-group analysis for the four OSMF studies reflected a significant reduction in I^2^ values from 76% to 51 % (P=0.11; Figure 2). 

**Figure 3:**
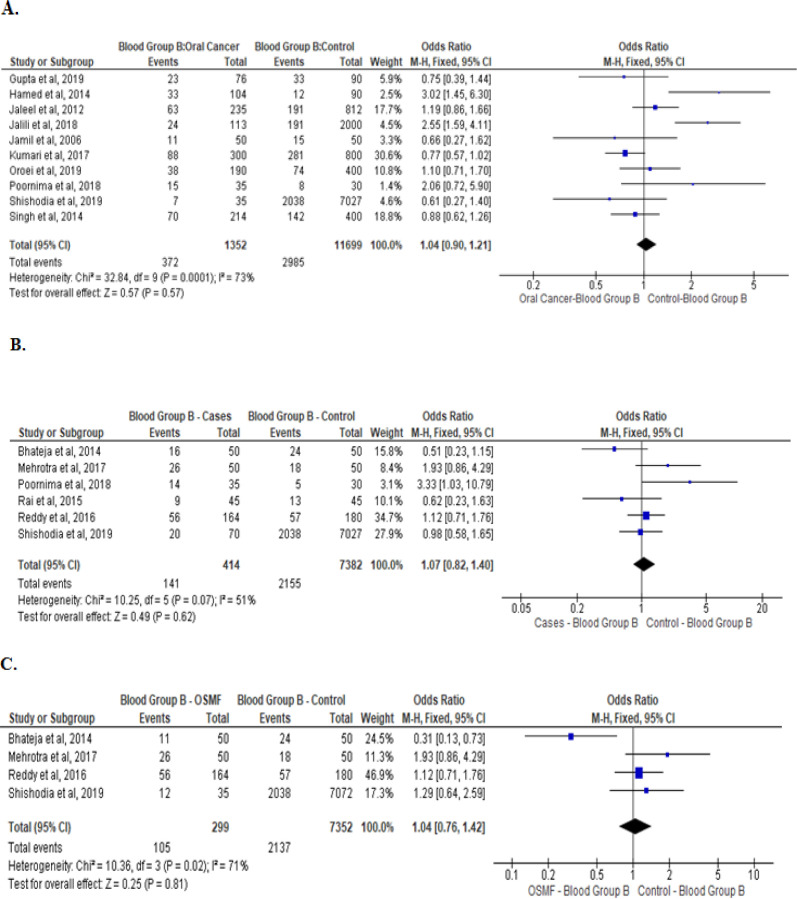
Type B Blood Group and Its Association between: Oral cancer patients with healthy controls (A) OPMD patients versus healthy controls (B), and OSMF patients versus healthy controls (C)

**Figure 4:**
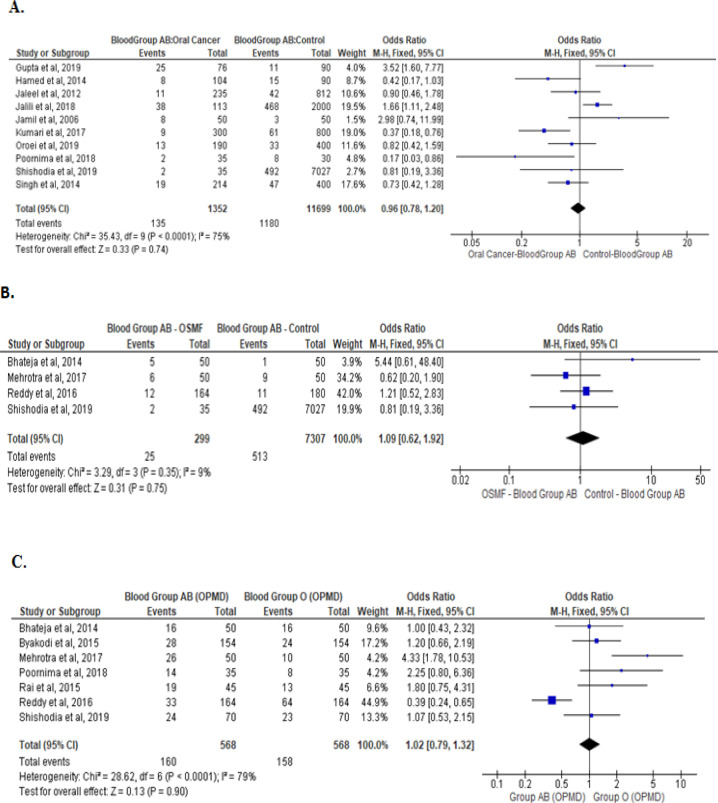
Type AB Blood Group and Its Association between: Oral cancer patients with healthy controls (A) OPMD patients versus healthy controls (B), and OSMF patients versus healthy controls (C)

**Figure 5:**
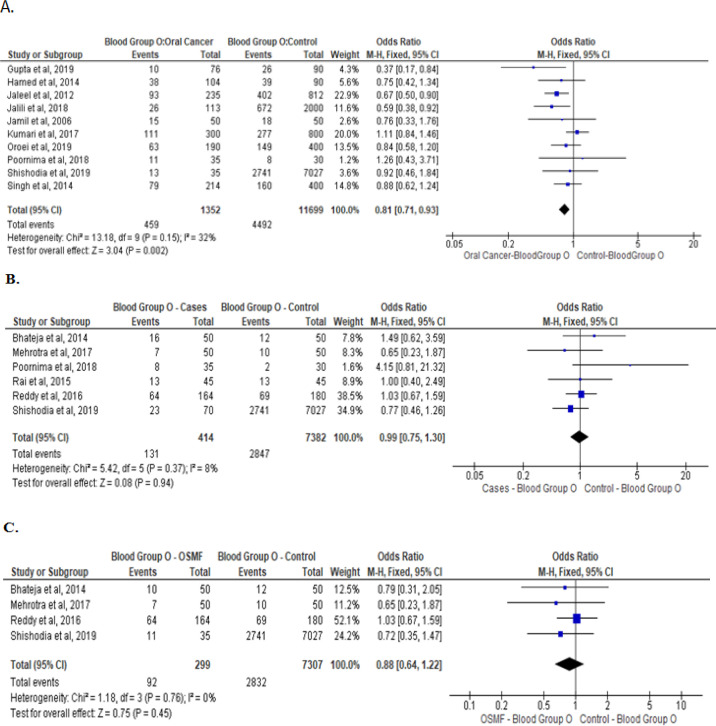
Type O Blood Group and Its Association between: Oral cancer patients with healthy controls (A) OPMD patients versus healthy controls (B), and OSMF patients versus healthy controls (C)

Meta-analysis of studies that evaluated the asociation of type B, AB, and O blood groups withoral cancer, OPMD, and OSMF are presented are presented in Figures 3, 4, and 5 respectively. No association was noted between blood group B and AB with oral cancer and OPMD. However, blood group O was significantly associated with lower chances of oral cancer (Odds ratio: 0.81[95% CI, 0.71, 0.93], P= 0.002; Figure 5). No such association was noted between blood group O with OPMD and OSMF.

The level of evidence obtained for the significant association of type A and type O blood group with oral cancer was moderate. Low level of evidence was achieved for the association between type A blood group and OPMD (Table 3).

**Figure 6:**
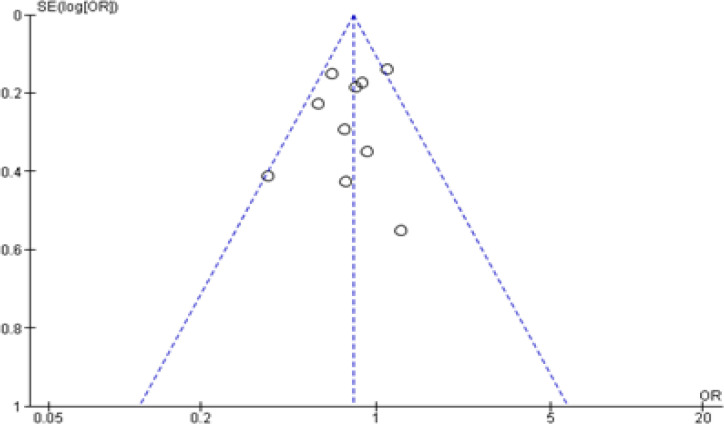
Funnel Plot of Included Studies Evaluating ABO Blood Group and Its Association with Oral Cancer

Funnel plots were drawn for evaluating the studies included in the meta-analysis. The vertical line of the funnel plot represents the pooled mean effect size, and the dotted lines represent the 95% confidence interval (Figure 6).

**Table 1:** Characteristics of Studies Included in Meta-Analysis

Study; Location	Study Design	Number of participants (n)	ABO blood grouping among Cases with Oral Cancer/OPMD/OSMF	ABO blood grouping among Controls with Oral Cancer/OPMD/OSMF	Significance of findings
Oral Cancer					
Gupta et al, 2019 New Delhi, India	Case control study (Six months in 2019)	Cases: 76 Control: 90	Type A - 18 (23.7%) Type B - 23 (30.1%) Type AB - 25 (32.9%) Type O - 10 (13.1%)	Type A - 20 (22.2%) Type B - 33 (36.7%) Type AB - 11 (12.2%) Type O - 26 (28.9%)	AB blood group associated with significantly higher chancers of oral cancer O Blood group associated with significantly lower chancer of oral cancer
Hamed et al 2014, Tehran, Iran	Case control study (2013)	Cases: 104 Control: 90	Type A - 25 (24%) Type B - 33 (32%) Type AB -8 (8%) Type O -38 (36%)	Type A - 24 (27%) Type B - 12 (13%) Type AB - 15 (17%) Type O -39 (43%)	B blood group associated with significantly higher chancers of oral cancer
Jaleel et al,2012 Karnataka, India	Case control study (2 months in 2010)	Cases: 235 Control: 812	Type A - 68 (29%) Type B - 63 (27%) Type AB - 11 (5%) Type O -93 (39%)	Type A - 177 (23%) Type B - 191 (23%) Type AB - 42 (5%) Type O -402 (50%)	A blood group associated with significantly higher chancers of oral cancer
Jalili et al, 2018 Tabriz, Iran	Case control study (2011 to 2014)	Cases: 113 Control: 2000	Type A - 45 (33.8%) Type B - 24 (18%) Type AB - 26 (19.5%) Type O - 38 (28.6%)	Type A - 699 (33.4%) Type B - 191 (23%) Type AB - 468 (23.4%) Type O - 672 (33.6%)	AB blood group had significantly higher chances of oral cancer O blood group associated with significantly lower chances of oral cancer
Jamil et al, 2006 Lahore, Pakistan	Case control study (2006)	Cases: 50 Control: 50	Type A - 16 (32%) Type B - 11 (22%) Type AB - 8 (16%) Type O - 15 (30%)	Type A - 14 (28%) Type B - 15 (30%) Type AB - 3 (6%) Type O - 18 (36%)	No association noted between ABO blood group and oral cancer
Kumari et al, 2017 Patna, India	Case control study (2 months in 2015)	Cases: 300 Control: 800	Type A - 92 (30.7%) Type B - 88 (29.3%) Type AB - 9 (3%) Type O - 111 (37%)	Type A - 181 (22.6%) Type B - 281 (35.1%) Type AB - 61 (7.6%) Type O - 277 (34.6%)	A blood group significantly associated with higher chances of oral cancer
Oroei et al, 2019 Iran	Case control study (2007 to 2017)	Cases: 190 Control: 400	Type A - 59 (32.9%) Type B - 38 (22.4%) Type AB - 13 (7.6%) Type O - 63 (37.1%)	Type A - 144 (36%) Type B - 74 (18.5%) Type AB - 33 (8.2%) Type O - 149 (37.3%)	No association noted between ABO blood group and oral cancer
Poornima et al, 2018 Telangana, India	Retrospective Case- Control study (2018)	Cases: 35 Control: 30	Type A - 6 (17.1%) Type B - 15 (45.7%) Type AB - 2 (5%) Type O - 11 (31.4%)	Type A - 9 (30%) Type B - 5 (16.6%) Type AB - 8 (26.7%) Type O - 8 (26.7%)	B blood group associated with significantly higher chances of oral cancer
Shishodiaet al 2019 Karnataka, India	Case control study (2013-2014)	Cases: 35 Control: 7027	Type A - 13 (37%) Type B - 7 (20%) Type AB - 2 (6%) Type O - 13 (37%)	Type A - 1757 (25%) Type B - 2038 (29%) Type AB - 492 (7%) Type O - 2741 (39%)	A blood group significantly associated with higher chances of oral cancer
Singh et al 2014, New Delhi, India	Case control study (6 months in 2013)	Cases: 214 Control: 7027	Type A - 46 (21.5%) Type B - 70 (32.7%) Type AB - 19 (8.87%) Type O - 79 (36.9%)	Type A - 51 (12.7%) Type B - 142 (35.5%) Type AB – 47 (11.7%) Type O - 160 (40%)	A blood group significantly associated with higher chances of oral cancer
Oral Potentially Malignant Disorders (OPMD)					
Bhateja et al, 2014 Pune, India	Case- Control study (2014)	Cases: 50 Control: 50	Type A - 36 (18%) Type B - 16 (8%) Type AB - 7 (3.5%) Type O - 16 (8%)	Type A - 13 (26%) Type B - 24 (48%) Type AB - 1 (2%) Type O - 12 (24%)	A blood group significantly associated with higher chances of OPMD
Mehrotra et al, 2017 Kanpur, India	Cross-sectional study with controls (2017)	Cases: 50 Control: 50	Type A - 11 (21%) Type B – 26 (52%) Type AB - 6 (13%) Type O - 7 (14%)	Type A - 13 (26%) Type B - 18 (35%) Type AB - 9 (19%) Type O - 10 (20%)	A and B blood group were associated with significantly higher chances of OPMD
Poornima et al, 2018 Telangana, India	Retrospective Case- Control study (2018)	Cases: 35 Control: 30	Type A - 8 (22.9%) Type B - 14 (40%) Type AB - 5 (14.9%) Type O - 8 (22.9%)	Type A - 9 (30%) Type B - 5 (16.6%) Type AB - 8 (26.7%) Type O - 8 (26.7%)	B blood group associated with significantly higher chances of OPMD
Rai et al, 2015 Karnataka, India	Cross-sectional study with controls (2015)	Cases: 45 Control: 45	Type A - 19 (42%) Type B - 9 (20%) Type AB - 4 (9%) Type O - 13 (29%)	Type A - 17 (38%) Type B - 13 (29%) Type AB - 2 (4%) Type O - 13 (29%)	No association noted between ABO blood group and OPMD
Reddy et al, 2016 Bhopal, India	Cross-sectional study with controls (2015)	Cases: 164 Control: 180	Type A - 33 (20.1%) Type B - 56 (34.1%) Type AB - 11 (6.7%) Type O - 64 (39%)	Type A - 42 (23.3%) Type B - 57 (31.7%) Type AB - 12 (6.7%) Type O - 69 (38.3%)	No association noted between ABO blood group and OPMD. Participants with blood group A were at higher risk of developing OSMF in comparison to others.
Shishodia et al, 2019 Karnataka, India	Case control study (6 months in 2013)	Cases: 70 Control: 7027	Type A - 24 (34.3%) Type B - 20 (28.6%) Type AB - 4 (2.8%) Type O - 23 (16.1%)	Type A - 1757 (25%) Type B - 2038 (29%) Type AB - 492 (7%) Type O - 2741 (39%)	No significant relationship noted between ABO blood group and OPMD

**Table 2:** Quality Assessment of Studies Included in the Meta-Analysis

Study Id	Selection of Participants	Confounding Variables	Measurement of Exposure	Incomplete Outcome Data	Selective Outcome Reporting
Gupta et al, 2019	L	M	L	L	L
Hamed et al, 2014	L	L	L	L	L
Jaleel et al, 2012	L	M	L	L	L
Jalili et al, 2018	L	M	L	L	L
Jamil et al, 2006	L	M	L	L	L
Kumari et al, 2017	L	M	L	L	L
Oroei et al, 2019	L	L	L	L	L
Poornima et al, 2018	L	M	L	L	L
Shishodia et al, 2019	L	M	L	L	L
Singh et al, 2014	L	M	L	L	L
Bhateja et al, 2014	L	M	L	L	L
Mehrotra et al, 2017	L	L	L	L	L
Rai et al, 2015	L	L	L	L	L
Reddy et al, 2016	L	M	L	L	L

L, Low risk of bias; M,: Moderate risk of bias; H, High risk of bias; U, Unclear risk of bias

**Table 3:** GRADE Assessment for Certainty of Evidence for Association between Blood Groups with Oral Cancer and OPMD

Certainty assessment							No of patients			
No of studies	Study design	Risk of bias	Inconsistency	Indirectness	Imprecision	Other considerations	Malnourished children	Well Nourished children	Relative (95% CI)	Certainty
Type A blood group and its association with oral cancer										
10	Observational studies	Serious	Not serious	Not serious	Not serious	Strong association, all plausible residual confounding would suggest spurious effect	388/1352 (28.6%)	3076/11699 (26.3%)	OR 1.27 (1.10 to 1.47)	MODERATE
Type 0 blood group and its association with oral cancer										
10	Observational studies	Serious	Not serious	Not serious	Not serious	Strong association, all plausible residual confounding would reduce the demonstrated effect	459/1352 (33.9%)	4492/11699 (38.3%)	OR 0.81 (0.71 to 0.93)	MODERATE
Type A blood group and its association with oral potentially malignant disorders (OPMD)										
6	Observational studies	Serious	Serious	Not serious	Not serious	Strong association, all plausible residual confounding would suggest spurious effect	131/414 (31.6%)	1851/7382 (25%)	OR 1.33 (1.01 to 1.75)	LOW

## Discussion

This meta-analysis is the first in scientific literature to assess the association between ABO blood groups and oral malignancy. Our meta-analysis included 14 trials and demonstrated a significant association between blood group A with oral cancer and OPMD. Blood group O was significantly associated with lower chances of oral cancer. No association was noted between blood group B and AB with oral cancer and OPMD. Also, no association could be found between ABO blood groups and OSMF.

Assessing the consistency of effects across studies is an essential part of a meta-analysis. The I² statistic describes the percentage variation across studies that are caused by heterogeneity rather than chance. Heterogeneity is classified into low (0-40%), moderate (30-60%), substantial (50-90%) and considerable (75-100%) heterogeneity, as reported in Cochrane Handbook 5.1 [31]. I^2^ values of 44% and 76% were noted for association of blood group A with oral cancer and OPMD. However, in sub group analysis I^2^ value reduced to 51% for association between OSMF and blood group A. I^2^ values of 32%, 8% and 0% were noted for association between blood group O and oral cancer, OPMD, and OSMF.

The quality of evidence, as qualified by GRADE, was moderate for the association between oral cancer with blood group A and O. Level of evidence was low for association between blood group A with other potentially malignant disorders due to inconsistency in results (I^2 ^=76%). Level of evidence was also moderate to low for lack of association between blood groups B and AB with oral cancer and OPMD.

Oral malignancy is a serious health problem resulting in high morbidity and mortality. This study demonstrates that there exists a relationship between ABO blood groups and oral cancer; specifically, blood group A and O. Health care providers in cancer screening and preventive programs should take these findings into considerations. Blood typing also can be used as a method to identify the susceptible individuals and counsel them.

World Health Organization predicts that global cancer deaths are projected to increase by 45% between 2008 and 2030 (WHO, 2020). Population projections by census bureau predict a 35% oral cancer incidence increase in United States by 2030 from the 2010 level (Smith et al., 2009). Oral cancer is largely curable if detected early and treated appropriately. Knowledge about the factors and conditions associated with oral cancer allows the construction of models to understand their development and to collaborate in the creation of evidence based public policies. 

We analysed, descriptive studies with controls, case control studies and retrospective studies with control together. However, although it is good practice to analyse data by study design, analysing for study design separately would have reduced the number of studies in each analysis to a number that impedes any meaningful conclusion. Several studies included in the analysis involved controls from large groups of blood donors. Even through these volunteer donors are considered to be representative of their study’s ethnic compositions, they may have other characteristics associated with altered risk of oral cancer. Limitations of the present study also include a limited number of analysed studies, and the study protocol was not registered. The present study was intended to fill the knowledge gap and to provide information and evidence for the association between ABO blood group system with oral cancer and other potentially malignant disorders. However, further prospective research on the association between blood groups with oral malignancy and potential malignant disorders is required.

In conclusion, this study demonstrates that there exists a relationship between ABO blood groups and oral cancer. Meta-analysis suggests A blood group has a greater risk for developing oral cancer and OPMD. Blood group O was associated with lower chances of oral cancer. No association was noted between ABO blood group system with OSMF.

## Author Contribution Statement

AS and BMP contributed equally to study design, study selection, data extraction, statistical analysis, draft preparation and approval of the final version.
